# A Comparison of Vitamin and Lutein Concentrations in Breast Milk from Four Asian Countries

**DOI:** 10.3390/nu12061794

**Published:** 2020-06-17

**Authors:** My Tuyen Thi Nguyen, Jieun Kim, Hyunjun Lee, Soyoon Won, Yongki Kim, Ji A. Jung, Dan Li, Xuan Hong Mai To, Khanh Trang Nguyen Huynh, Thanh Van Le, Beenish Israr, Hyun Joo An, Jaehan Kim

**Affiliations:** 1 Department of Food and Nutrition, Chungnam National University, Daejeon 34134, Korea; mytuyen1108@gmail.com (M.T.T.N.); ai0107380@gmail.com (J.K.); dlguswns122@gmail.com (H.L.); elementyoon@gmail.com (S.W.); 2 College of Agriculture, Can Tho University, Can Tho City 900000, Vietnam; 3 Central Research Laboratory, Maeil Co., Ltd., 63 Jinwiseo-ro, Jinwi-myeon Pyeongtaek, Gyeonggi-do 17706, Korea; kim6767@maeil.com (Y.K.); jungjia@maeil.com (J.A.J.); 4 College of Food Science and Engineering, Changchun University, Changchun 130022, China; drlidan@yahoo.com; 5 Department of Obstetrics and Gynecology, University of Medicine and Pharmacy at Ho Chi Minh City, Ho Chi Minh City 700000, Vietnam; tomaixuanhong@ump.edu.vn (X.H.M.T.); tranghnk08@gmail.com (K.T.N.H.); 6 Faculty of Nursing and Medical Technology, University of Medicine and Pharmacy at Ho Chi Minh City, Ho Chi Minh City 700000, Vietnam; thanhvan.vatlytrilieu@gmail.com; 7 Faculty of Food, Nutrition and Home Science, University of Agriculture, Faisalabad 38000, Pakistan; beenish_israr@hotmail.com; 8 Graduate School of Analytical Science and Technology, Chungnam National University, Daejeon 34134, Korea; hjan@cnu.ac.kr

**Keywords:** human milk, quantification, vitamin, liquid-chromatography, mass spectrometry

## Abstract

Vitamins are the essential elements for human life and, particularly, for infant health. Human milk is the best source of nutrients for newborns, however, the information of vitamins in Asian maternal milk is still limited. In this study, we have collected 580 Asian maternal milk samples from Korea (*n* = 254), China (*n* = 137), Pakistan (*n* = 92), and Vietnam (*n* = 97). The vitamin concentrations, including vitamin B-groups (8 vitamins), fat-soluble vitamin (retinol, D, E, K) and lutein in the breast milk of were investigated. The concentration of thiamin (B_1_), biotin (B_7_), and folic acid (B_9_) in mother’s milk of four countries were not considerably different, while riboflavin (B_2_), pantothenic acid (B_5_), and pyridoxine (B_6_) level in Vietnam samples were significantly lower than those in other countries. In contrast, retinol (A) and tocopherol (E) were found to be higher levels in Vietnamese maternal milk. Korean and Chinese maternal milk had low concentrations of retinol that may cause vitamin A deficiency in children. However, Chinese mother’s milk was distinguished with a high concentration of lutein. Pakistani mother’s milk was observed as having a significant problem of folic acid (B_9_) deficiency. Regardless of the country, vitamin B_12_, K, and D did not seem to be provided sufficiently through maternal milk. The moderate positive correlations were found between vitamin concentrations in each country and the pooled sample. The data obtained in this study were able to provide vital information to assess the nutritional status of breast milk in Asian countries and contributed to the efforts of ensuring the best nutrition for Asian children.

## 1. Introduction

In recent years, the attention to the concept of early-life metabolic programming and the future health of growing infants was remarkably rising. Early environmental factors, such as chemical exposure, diet, and nutritional supplements might have after-effects on human biology and long-term health. Human milk is recommended as the best source of nutrients for infants from 0 to 6 months [1,2]. It provides not only metabolic nutrients, but also functional elements such as vitamins, minerals, oligosaccharides, and various protective factors that could give long-term effects on physical, mental [3] and immune system development [1,2,4,5].

Among these functional compounds, vitamins, particularly, have fundamental roles for the proper growth and development of infants. Vitamin A is one of the most important micronutrients affecting vision, the immune system, lung development, and maturation [6,7,8]. Vitamin D is involved in the calcium absorption, mineralization of the skeleton, and the prevention of rickets in children [9,10,11,12]. Vitamin E is a strong antioxidant that inhibits lipid peroxidation and protects cell membranes and lipoproteins from free radicals [8]. The water-soluble vitamin B complex is a co-enzyme of numerous biochemical reactions and has various functions in the human body [13,14,15,16]. In addition, lutein is a major carotenoid in the human eye [17]. It is also a dominant carotenoid in the infant’s brain as well, and plays an important role in cognitive development [18]. 

It has been well noticed that the deficiency of vitamin or lutein could lead infants to improper development, diseases, and even cause death [5,19,20]. Vitamin deficiency of an infant occurs due to their limited amount of vitamin reserves at birth and the rapid consumption during their early age of growth. Since vitamin supplement depends only on the exogenous source, the amount of vitamins in human milk was essential for infant health and development. Moreover, the concentration of retinol in human milk is also used as a biomarker of vitamin A deficiency in lactating women and children up to 71 months [21]. 

The amount and types of vitamins and lutein in human milk are closely related to the mother’s diet, nutritional status, and lactation duration [22,23]. Up to now, vitamin contents in breast milk were studied in several developed countries, however, the information about vitamin status in Asian mother’s milk is still rare. Moreover, the different analytical methods contribute to the strong variation of vitamin content between countries. Here, we attempt to collect mother’s milk from four Asian countries in different climate zones, including the temperate zone (Northeastern China), sub-tropical zone (South-Korea), and tropical zone (Vietnam and Pakistan). These mothers also consumed different styles of food, such as Muslim food in the Pakistan population and Asian cuisine for the others. Vitamin concentrations in human milk were studied and compared between countries.

## 2. Materials and Methods 

### 2.1. Sample Collection

Human milk samples were collected from Korea, China, Pakistan, and Vietnam from 2017 to 2018. This study was approved by the Institutional Review Board of Chungnam National University (Korea), Maeil Dairies. Co., University of Medicine and Pharmacy at Ho Chi Minh City (Vietnam) and University of Agriculture (Faisalabad, Pakistan). Informed consent was obtained from all participants and methods were performed in accordance with the relevant guidelines and regulations. In this study, the number of human milk samples from Korea, China, Vietnam, and Pakistan were 254, 137, 92, and 97 samples, respectively. 

Human milk was directly collected in a sterilized 50 mL conical tube (Corning, NY, USA), by hand press or breastmilk pump. The total volume of maternal milk donated from each mother was 50–150 mL. Then, the sample was delivered to the laboratory with a gel ice pack in well-insulated containers, to maintain a low temperature. The human milk sample was stored at −80 °C before analysis. The sample was rejected from analysis if the storage time was longer than 2 months.

### 2.2. Materials

Vitamin standards that include thiamin hydrochloride, riboflavin, nicotinic acid and nicotinamide, calcium-D-pantothenate, pyridoxin-hydrochloride, biotin, folic acid, cyanocobalamin and phylloquinone, were purchased from AccuStandard, New Haven, CT, USA. The following standards and reagents were obtained from Sigma-Aldrich, (Seoul, Korea): pyridoxal-hydrochloride (B_6_), all-*trans* retinol, all-*trans* tocopherol (E), ergocalciferol (D_2)_, cholecalciferol (D_3_), all-*trans* lutein, butylated hydroxytoluene (BHT), HPLC grade solvent (methanol, ethanol, acetonitrile, and hexane). The HPLC grade MTBT (tert-Butyl methyl ether) was purchased from Thermo Fisher, Korea. Moreover, 7-dehydrocholesterol (DHC) was purchased from Cayman Chemical (MI, USA). Potassium hydroxide, ascorbic acid and sodium chloride were purchased from Deajung. Co. Ltd. (Busan, Korea).

### 2.3. Water-Soluble Vitamin Analysis

#### 2.3.1. Sample Preparation

The milk sample (0.5 mL) was mixed with 1 mL of ethanol and sonicated for 20 min. Then, 2 mL of hexane (containing 0.025% BHT) was added to the sample and shaken for 20 min. The mixture was centrifuged (3600× *g*, 15 min). The aqueous layer was collected and mixed with 0.5 mL ice ethanol (−80 °C, 1 h). The supernatant obtained after centrifugation (21,000× *g*, 20 min) was quickly evaporated to remove ethanol. The sample was cleaned up by solid phase extraction using C_18_ cartridge (Sep-Pak C_18_, 200 mg sorbent, Waters, UK). The cartridge was activated and conditioned by methanol and distilled water. After sample loading, water-soluble vitamins were eluted by 6 mL of methanol: water (1:1). The elution was completely evaporated at 37 °C. The sample was reconstituted and diluted in acetonitrile: water (3:7) solution (containing 0.1% formic acid) before the injection into LC-MS/MS. The number of human milk samples in China, Korea, Pakistan and Vietnam were 111, 155, 97 and 92, respectively.

#### 2.3.2. LC-MS/MS Conditions

Vitamin B-complexes were analyzed by using ultra performance liquid chromatography tandem mass spectrometry (6460 Triple Quadrupole System, Agilent, CA, USA). The analytical column was Glycan BEH Amide (2.1 × 100 mm, 0.35 mm, Waters, Hertfordshire, UK) and was kept at 30 °C during analysis. The solvents were 50% acetonitrile (solvent A) and 90% acetonitrile (solvent B), each of which contained 0.1% formic acid and 10 mM ammonium formate. The flow rate was 0.3 mL/min and the gradients between the time points were as follows: 0–2 min, B = 99.9%; 2–6 min, B = 99.9–70% B; 6–12 min B = 70–30%; 12–14 min, B = 30–10%; 14–20 min, B = 10%; 21–30 min, B = 99.9%. The MS was performed in positive mode with the MS conditions, as follows: gas temperature, 300 °C; gas flow, 8 L/min; nebulizer pressure, 45 psi; sheath gas temperature, 300 °C; sheath gas flow, 12 L/min; capillary voltage, 4000 V (positive); nozzle voltage, 1000 V (positive). The injection volume was 1 μL. The MS/MS parameter and MRM transitions of analytes are summarized in Table S1.

The standard curves were set up with the solution of thiamin, riboflavin, nicotinic acid, pyridoxine, pyridoxal ranged from 0.1 to 200 µg/L. The standard solution of pantothenic acid, nicotinamide ranged from 50 to 5000 µg/mL and 0.1 to 500 µg/mL, respectively. The *r*^2^ of the standard curve for all standard materials ranged from 0.994 to 0.999. The limit of quantitations (LOQ) for all vitamins were 0.1–0.6 ppb, except vitamin B_9_ (4.5 ppb) and B_12_ (1.8 ppb).

### 2.4. Fat-Soluble Vitamins and Lutein Analysis

#### 2.4.1. Samples Preparation

The milk sample (2 mL) was put into a 15 mL centrifuge tube. Then, 4 mL of ethanol (0.1% (*w*/*v*) BHT), 1 mL of NaCl solution (2%, *w/v*), 1 mL of ascorbic acid solution (4%) and 1 mL of KOH solution (60%, *w/v*) were subsequently added. The mixture was saponification at 70 °C for 60 min in shaking water-bath. After that, sample was quickly cooled in ice water. The sample was double extracted in 5 mL hexane. Both hexane fractions were combined and washed with NaCl (0.1%) to remove KOH. Then, hexane was evaporated under vacuum condition (40 °C, 30 min). The residual was reconstituted in 100 µL of isopropanol: hexane (75:25, 0.025% BHT). The sample was filtrated through a 0.2 µm and injected into HPLC. 

#### 2.4.2. HPLC Conditions

Fat-soluble vitamin was quantified by HPLC coupled with UV detector (1260 Agilent, California, USA). Analytes were separated using a C30 YMC Carotenoids column (5 µm, 4.6 × 250 mm, YMC Korea Co. ltd., Gyeonggi-do, Korea) at 30 °C. The mobile phase A consisted of methanol: acetonitrile: water (4:5:1) and mobile phase B was methyl *tert*-butyl ether. The flow rate of the mobile phase was 1 mL/min. The gradient profile was as follows (*t* in min): *t*_0_, B = 5%; *t*_20_, B = 25%; *t*_25_, B = 40%; *t*_29_, B = 70%; *t*_33_, B = 90%; *t*_39_, B = 90%; *t*_40_, B = 5%; *t*_50_, B = 5%. Fat-soluble vitamins were detected at various wavelengths: retinol: 325 nm, vitamin D: 265 nm, vitamin E: 220 nm, vitamin K_1_: 246 nm, and lutein: 445 nm. The limit of quantitation (LOQ) of the method was 0.6–2.7 ppb for all fat-soluble vitamins, except vitamin E (10.5 ppb).

### 2.5. Statistical Analysis

The data were expressed as mean ± standard deviation. The one-way ANOVA and Scheffe’s multiple range test (*p* < 0.05) were used to define the significant difference between groups. The correlation between variables was determined by Pearson’s product-moment correlation coefficient (*r*-value). Stata/SE (version 12.1) has been used for the statistical analysis and the generation of resulting figures. 

## 3. Results

### 3.1. Variation of Vitamin Concentrations in Human Milk between Countries

#### 3.1.1. Water-Soluble Vitamins

The concentrations of water-soluble vitamin in human milk were diverse between and within the countries. In general, niacin (nicotinic acid + nicotinamide), pantothenic acid, retinol, and tocopherol were relatively abundant in human milk (0.4–0.6, 1.2–2.6, 0.3–0.8, 1.9–4.5 g/L, respectively). Thiamin, riboflavin, pyridoxine, biotin, phylloquinone, and lutein concentrations ranged from 1–150 µg/L (Table 1). Not all the vitamin B complexes were found from human milk samples. Vitamin B_9_ (folic acid) were observed 41.1% of Vietnamese mothers milk, meanwhile, only 2% of Pakistani mothers milk contained the folic acid above the LOQ (4.5 µg/L). Vitamin B_12_ occurred in a low quantity in human milk as well. Only 2–23% of samples in each country had vitamin B_12_ higher than LOQ (1.8 µg/L). The average and the standard deviation in Table 1 were calculated using the values above the LOQ. 

**Figure 1:**
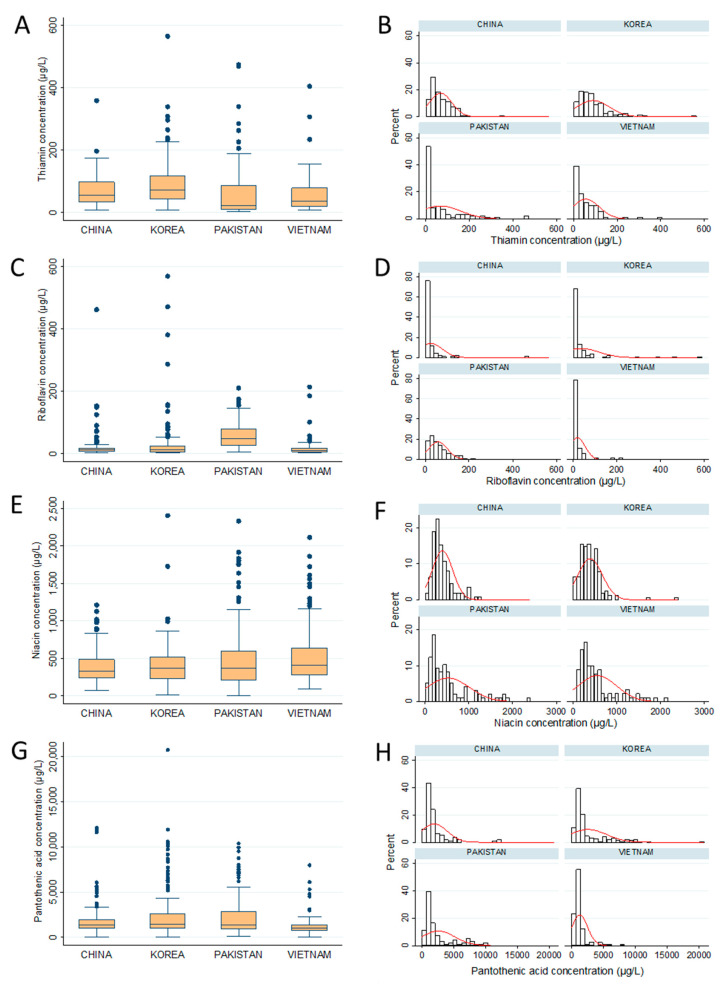

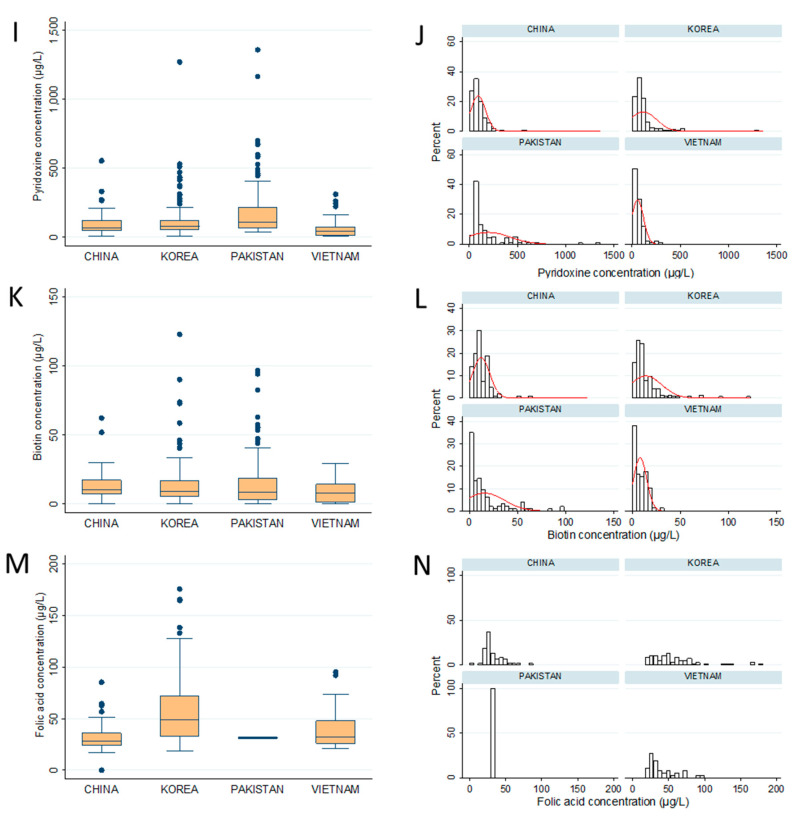
Distribution of water-soluble vitamins in mother’s milk of the four countries by box plot and histograms. In the box plot, box indicates the first (Q1) and third (Q3) quartile, and the Whiskers indicate the ±1.5× interquartile ranges from the box. The line in the box represent the media value. The red line in the histogram represents the normal distribution by computer simulation. (**A**,**B**): Thiamin; (**C**,**D**): Riboflavin; (**E**,**F**): Niacin: (**G**,**H**): Pantothenic acid; (**I**,**J**): Pyridoxine; (**K**,**L**): Biotin; (**M**,**N**): Folic acid.

Median and the total average that included the zero concentration (<LOQ) were summarized in Supplementary Table S2. The comparison of vitamin concentration by country was described in both box plot and histogram (Figure 1). Regardless of countries, numerous outliners were obtained, showing a wide personal variation, even within the same country.

• Thiamin (Vitamin B1)

The average of free thiamin in Asian mother’s milk ranged from 50 to 90 µg/L. Chinese and Korean maternal milk exhibited less personal variation, whereas, around 55 and 40% of Pakistani and Vietnamese maternal milk had low thiamin content less than 20 µg/L. A wide personal variation of thiamin in Pakistani maternal milk was observed, showing RSD of 140.9% (Figure 1A,B).

• Riboflavin (Vitamin B_2_)

In this study, riboflavin was detected in 73%, 78%, 83%, and 100% of human milk from Korea, Vietnam, China, and Pakistan, respectively (LOQ: 0.3 µg/L). The average riboflavin concentrations were 20–60 µg/L (Table 1). Pakistani mother’s milk had the highest concentration of riboflavin (58.0 ± 43.3 µg/L), which were 1.6–3 times greater than in the maternal milk of other countries. Statistically, there was no significant difference in the average riboflavin content of Korean (36.1 ± 82.8 µg/L), Chinese (25.3 ± 53.5 µg/L), and Vietnamese (19.9 ± 34.7 µg/L) maternal milk. However, numerous outliners were observed from the Korea, China, and Vietnam populations, which resulted in the strong variation of riboflavin concentration in human milk (Figure 1C,D). 

• Niacin (Vitamin B_3_)

Niacin concentration was estimated by the sum of niacinamide and nicotinic acid content. Niacin levels in human milk from four Asian countries were similar (Figure 1E,F) and the medians were almost the same (Table S2). However, ~15% (*n* = 14) of samples in the Vietnam and Pakistan population had an extremely high niacin concentration over 1000 µg/L. Subsequently, the average of niacin in Pakistani and Vietnamese maternal milks (523.9 ± 485.2 and 553.8 ± 440.2 µg/L, respectively) seemed to be higher than those in Chinese and Korean maternal milk, which were about 400 µg/L. 

• Pantothenic acid (Vitamin B_5_)

Pantothenic acid was the most abundant water-soluble vitamin, which ranged from 1200 to 2500 µg/L. Vietnamese maternal milk had the lowest concentration of pantothenic acid, with only 1266.1 ± 1242.7 µg/L. It was nearly half of those in Korea and Pakistan, and lower than China. The pantothenic acid concentration in Asian maternal milk was widely spread from 0.01 to 15 g/L. Meanwhile, the Vietnam sample had a less personal variation, showing that 75% of samples were lower than 1.5 g/L (Figure 1G,H).

• Pyridoxine (Vitamin B_6_)

Pyridoxal is the predominant form of vitamin B_6_ in mother’s milk. In this study, the pyridoxine content (vitamin B_6_) was calculated as total pyridoxine and pyridoxal concentration in milk. In Asian maternal milk, pyridoxine ranged from 56 to 200 µg/L. There was no statistical significant difference among the average of pyridoxine content of Korean, Chinese, and Vietnamese mother’s milk (Table 1). Meanwhile, the highest concentration of pyridoxine was found in Pakistani mother’s milk 196.7 ± 225.3 µg/L which was almost 4 times as many as those in Vietnam (56.4 µg/L). Around 20% of samples in the Pakistan population exhibited a high concentration from 300 up to 1500 µg/L (Figure 1I,J). 

• Biotin (Vitamin B_7_)

The biotin concentrations in human milk were similar between four countries, however, a statistical difference has been found in their average concentrations. Biotin concentrations of Chinese, Korean, and Pakistani maternal milk were in the same range of 12.1–15.8 µg/L with the high value outliers. Meanwhile, biotin in Vietnamese human milk was at a relatively low level (8.1 µg/L), without the high value outliers (Figure 1K,L). 

• Folic acid (Vitamin B_9_)

In this study, only the free form of folic acid was quantified as a vitamin B_9_. Many human milks did not contain folic acid, in general. It has been found that only 40–60% of samples from each country have the µg/L level of LOQ. Particularly, only 2% of Pakistani mother’s milk contained folic acid above the quantitation limits. The concentration of folic acid in human milk, if presented, ranged from 30 to 60 µg/L (Figure 1M,N). 

• Cyanocobalamin (B_12_)

Vitamin B_12_ was found in human milk at a low quantity. Approximately, 25% of Pakistani mother’s milk had cyanocobalamin above the LOQ (1.8 ppb), while it was only 2–13 percent in Korean, Chinese, and Vietnamese. The average concentrations ranged from 2 to 8 µg/L and were not significantly different between countries.

#### 3.1.2. Fat-Soluble Vitamins

Fat-soluble vitamins in human milk, including retinol, vitamin D, E, K, and lutein, were quantified by UV-HPLC (Table 2). Vitamin E was the richest among fat-soluble vitamin, showing the concentration of 2–4 mg/L. Retinol was the second abundant and occurred in human milk from 300–900 µg/L. Vitamin D concentration in all samples was under the limit of detection of our method (Vitamin D_2_ = 0.5 ppb and D_3_ = 1 ppb). Only 40–70% of mother’s milk in each country had vitamin K (phylloquinone) above the LOQ (<2.7 ppb). 

• Retinol (Vitamin A)

**Figure 2:**
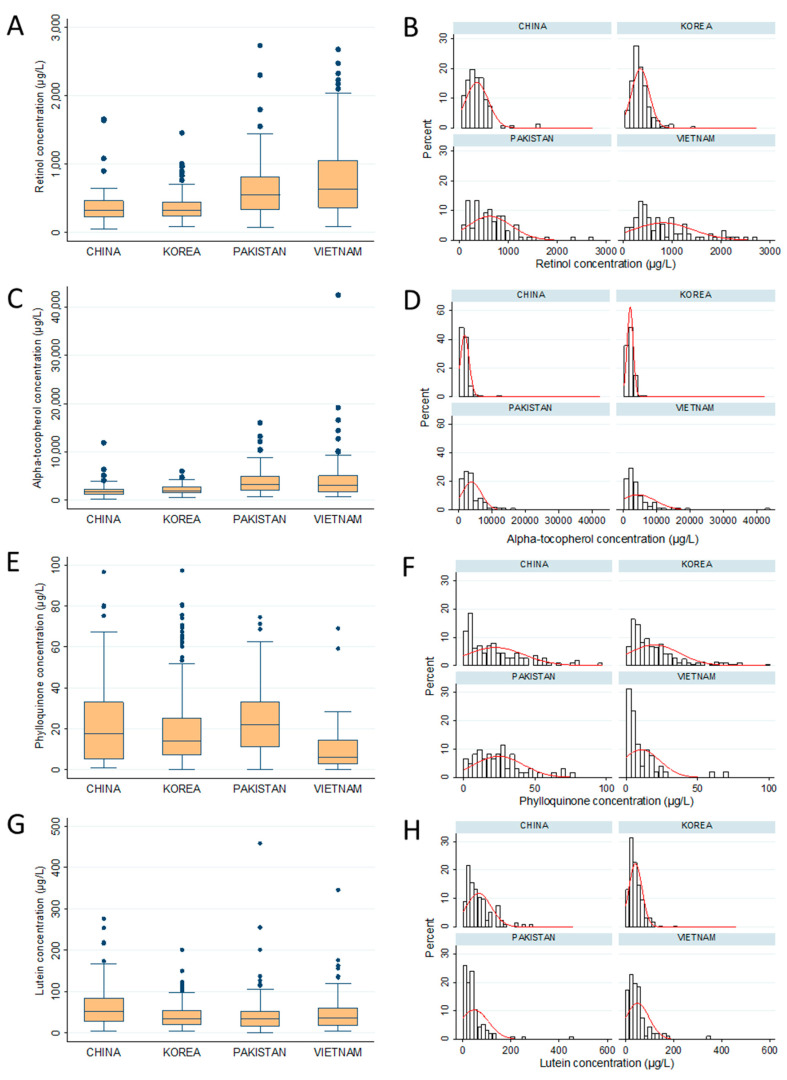
Distribution of fat soluble vitamin in mother’s milk of the four Asia countries. In the box plot, box indicates the first (Q1) and third (Q3) quartile, and the Whiskers indicates the ±1.5× interquartile ranges from the box. The line in the box represents the media value. The red line in the histogram represents the normal distribution by computer simulation (**A**,**B**): Retinol; (**C**,**D**): Apha-tocopherol; (**E**,**F**): Phylloquinone; (**G**,**H**): Lutein.

In human milk, retinol present in free and ester form together. In this study, retinyl esters were hydrolyzed to release free retinol by saponification before HPLC analysis. Total retinol concentration in Chinese and Korean maternal milk were similar together (350–360 µg/L), but significantly lower than that in Pakistani (622.1 ± 447.3 µg/L) or Vietnamese (813.6 ± 609.0 µg/L). Interestingly, retinol concentration in the Vietnamese mother’s milk was 2.3 times higher than Korean and Chinese and exhibited strong personal variation. The distribution retinol in human milk of Korea and China were similar to normal distributions, although several high concentration outliers were observed (Figure 2A,B). Meanwhile, Pakistan and Vietnam data spread out widely from 70 to 2700 µg/L, with the relative standard deviation (RSD) of 72–75%.

• Tocopherol (Vitamin E)

Similar to retinol, the concentration of breast milk tocopherol in the four countries can be divided into two groups: low-narrow distribution (China and Korea) and high-wide distribution (Vietnam and Pakistan) (Figure 2C,D). The average of vitamin E in Pakistani and Vietnamese mother’s milk were 3.9 and 4.4 g/L, respectively. China and Korea milk samples were ~2 mg/L, nearly half (40–50%) of those in Pakistan or Vietnam.

• Phylloquinone (Vitamin K)

Phylloquinone presented as a low quantity in human milk, and varied from 0 to 97.4 µg/L. About 60–70 percentage of Chinese, Korean, and Pakistani maternal milk had vitamin K more than 2.7 µg/L (LOQ). However, only 44% of the Vietnam population had phylloquinone concentration above LOQ. The average vitamin K concentration of Korea and China samples were 13–19 µg/L with RSD of 86–97%. Pakistani mother’s milk contained 25.7 ± 17.2 µg/L of phylloquinone, which was significantly greater than those in Vietnam (12.9 ± 13.9 µg/L). The distributions of phylloquinone level in human milk were similar between four countries (Figure 2E,F).

• Lutein

Basically, the distribution patterns of lutein concentration in the breast milk of all four countries were similar (Figure 2G,H). The average lutein concentration in human milk did not significantly differ among the Korea, Pakistan, and Vietnam populations (41.3, 47.5, and 49.7 µg/L, respectively). It is noticed that the higher concentration of lutein was observed in Chinese maternal milk (66.1 µg/L).

• Vitamin D

Vitamin D, including ergocalciferol (vitamin D_2_) and cholecalciferol (vitamin D_3_), was measured in human milk by UV-HPLC. The concentration of vitamin D_2_ and D_3_ in human milk of all four countries was lower than the limit of detection of D_2_ = 0.5 µg/L and D_3_ = 1µg/L. However, cholesterol, which is the precursor of vitamin D_3_, was detected in human milk at the level of 90–175 mg/L. Moreover, desmosterol, a precursor of cholesterol, was also observed in human milk, with the concentration ranging from 10–13 mg/L.

### 3.2. Correlation between Vitamins and Lutein 

Pearson’s correlations between vitamins in the pooled sample were illustrated in Table 3. Most of the correlation coefficients are low (<0.5). There were several moderate positive correlations observed, including pyridoxine (B_6_) versus thiamin (B_1_) and pantothenic acid (B_5_) (r = 0.450, 0.464); retinol versus tocopherol (0.497).

In the comparison of each individual country, the correlations between components were quite random and the similarity was not found among four countries. However, there were several common features: correlation is found among vitamin B together or fat-soluble vitamins together. Positive correlations were observed such as vitamin B_6_ versus vitamin B_5_ in China, Pakistan and Vietnam (*r* = 0.61, 0.631, 0.479); vitamin B_6_ versus vitamin B_2_ in China, Korea, and Vietnam (*r* = 0.704, 0.479, 0.604); retinol versus vitamin E in China and Pakistan (*r* = 0.461; 0.795). Other specific correlations in each country could be seen in Tables S3–S6.

## 4. Discussion

Vitamins belong to micronutrients and are essential for infant growth and development. Breast milk contains almost all the vitamins that a healthy full-term baby needs, although the supplementation of vitamin D and K is still recommended for infants. However, not all babies can be breastfed, for various reasons, which makes infant formula inevitable. To supply sufficient and balanced vitamins for infant’s needs, the reference information of vitamin concentration in human milk is necessitated for infant formula setting up.

Vitamin concentrations in breast milk were not consistent widely in many publications. It is partly due to the complex structures and isomers of vitamins and the subsequent diversity of the numbers and types of vitamin standards. Vitamin B occurs in human milk under various forms and derivatives. It was reported that vitamin B_1_ is present in human milk under the forms of free thiamin, thiamin-monophosphate, and thiamin-pyrophosphate. Similarly, vitamin B_2_ was founded as free riboflavin and flavin adenine-dinucleotide [19], or vitamin B_3_ naturally presented as nicotinic acid and nicotinamide in human milk. Vitamin B_6_ mainly occurs as pyridoxal and a small amount of pyridoxine + pyridoxamine [24]. Vitamin B_9_ in human milk consisted of unmetabolized folic acid (23%), 5-methyl-tetrahydrofolate (55%), and other reduced folates (tetrahydrofolate (THF), 5-formyl-THF and 5, 10-methenyl-THF) [25], and so on. In the same way, the fat-soluble vitamin also had various forms and isomers, such as vitamin E (alpha, beta, and gamma-tocopherol), vitamin K (phylloquinone, menaquinones) [26], vitamin D metabolites [21], *cis* and *trans*-lutein isomer [27]. 

In this study, thiamin (free form), riboflavin (free form), niacin (nicotinamide and nicotinic acid), D-pantothenic acid, vitamin B_6_ (pyridoxal and pyridoxine), biotin, folic acid (free form) and cyanocobalamin were analyzed. Although the concentrations of all vitamin metabolites were not able to be covered in one study, we attempt to make a comparison of our human milk vitamin data obtain from four Asian countries and previously published data of the same metabolites (Table 4). Apparently, each vitamin concentration found from various studies varied in the wide range. On the other hand, the level of vitamin obtained in this study was reasonable and within a similar range of previous studies. In this study, human milk was randomly collected during the lactation stage, from both supplementation users and non-users. Vitamin K value in this study was quite high, however, it should be noted that the value was only calculated from the sample observed the vitamin K (LOQ = 2.7 µg/L). There was 30 to 54% of mother’s milk in which vitamin K was not found. Taking these samples into consideration, the concentration of vitamin K in Asian human milk became 12.6 ± 17.0 µg/L (median: 6.0 µg/L). 

Although we could not cover all of the vitamins, a general comparison of vitamin concentration in human milk between Asian countries would be valuable. The vitamin B level in the Vietnamese population was usually significantly lower than those in breast milk from other countries, particularly, riboflavin (B_2_), pantothenic acid (B_5_), and pyridoxine (B_6_). Pakistani maternal milk was noticed with a low concentration of folic acid. It was reported that thiamin, riboflavin, and pyridoxine in the mother were also strongly influenced by the mother’s diet or supplementation [15,28]. Hence, a recommendation of supplementation or improving vitamin B in the diet seems to be required for Vietnamese and Pakistani mothers. 

Breastmilk is a great source of lutein for infants. The lutein concentration in breastmilk may vary from 10 to 100 μg/L, which is several times higher than that in cow milk (approximate 5–15 μg/L) [29]. Particularly, lutein originating from breast milk plays an important role in visual processing in early life [30]. It is the predominant carotenoid in adults and infant brains [31], especially in the neocortex area, and also a key functional component in the neural retina [32]. Recently, research on rhesus macaques indicated that lutein supplementation of infant formula significantly increased serum and tissue lutein concentrations compared to the unsupplemented formula, however, both of them were still lower than those in breastfed infants. 

Interestingly, lutein in Chinese maternal milk was higher than in other countries. The average of lutein concentrations in human milk from Korean, Pakistani, and Vietnamese mothers was 40–50 µg/L while 66.1 µg/L in Chinese maternal milk. It was also observed that lutein in China maternal milk had a high content, up to the median of 93.1 µg/L [33]. It might be due to the difference in the mother’s diet, since the breast milk lutein concentration depended on maternal intake [34]. 

Based on the data we observed, it was still difficult to conclude whether the human milk of Asian mothers could provide enough vitamin for their baby or not. However, the data in this study also clearly indicates that vitamins B_12_, K, and D were not sufficient in human milk. Vitamin K intake for the first 0–6 months is recommended at 5 mg/day [51]. Based on the obtained data, approximately 43–53% of infants in Korea, China, and Pakistan and 75% of Vietnam did not get enough vitamin K. Vitamin B_12_ and D levels of other countries were also reported as low as the range of pmol/L [35,43]. 

Under the circumstances, providing additional vitamin B_12_ and D through the mother’s diet or supplements seems to be necessary. It was reported that B_12_ supplementation of the mother during pregnancy and early lactation could improve vitamin B_12_ status in breast milk and infant [37]. Moreover, the mother supplemented with a high dose of vitamin D was able to increase a slight amount of vitamin D in her breast milk [43,52]. In this regard, the WHO strongly recommended giving vitamin supplementation for breastfed infants [51]. It is recommended that they supplement with 400 IU per day of vitamin D, beginning in the first few days of life [53]. Additionally, it has been recommended that mothers should pay more attention to their diets or provide vitamin K supplementation to their baby [51].

Vitamin A deficiency is one of the most common health problems in the world. In addition, retinol concentration of mother’s milk of less than 1.05 μmol/L is used as a biomarker of vitamin A deficiency (VAD) in lactating women and children up to 71 months [51]. The percentage of the indicator (milk retinol <1.05 μmol/L) ≥25%, from 10 to 25%, or <10%, is considered as severe, moderate, and mild VAD, respectively [51]. Interestingly, retinol in Vietnamese maternal milk was richer than in other countries. The Korean and Chinese populations had a high percentage of milk retinol <1.05 µmol/L, with 44%, followed by Pakistan (23%) and Vietnam (16%). These data indicated that Korea and China mother and infant are at high risk of VAD. 

A comprehensive comparison of fat and water-soluble vitamins in breast milk between countries has been carried out in this work. The data exhibited a general view of vitamin status in Asian maternal milk. It could provide useful information for mother and baby care that may be used as a reference to establish the best infant formula for Asian babies. 

## Figures and Tables

**Table 1:** Average of water soluble-vitamin concentration in human milk (µg/L).

	CHINA	KOREA	PAKISTAN	VIETNAM
No. Sample	111	155	97	92
Thiamin (B_1_)	68.1 ± 51.2 ^a,b^	89.3 ± 74.3 ^b^	66.5 ± 93.7 ^a,b^	56.5 ± 61.5 ^a^
Riboflavin (B_2_)	25.3 ± 53.5 ^a^ (82.9%)	36.1 ± 82.8 ^a,b^ (72.9%)	58.0 ± 43.3 ^b^ (100%)	19.9 ± 34.7 ^a^ (77.8%)
Niacin (B_3_) ^i^	396.7 ± 233.3 ^a^	393.7 ± 278.3 ^a^	523.9 ± 485.2 ^a,b^	553.8 ± 440.2 ^b^
Pantothenic acid (B_5_)	1924.0 ± 2047.4 ^a,b^	2571.2 ± 2932.2 ^b^	2557.4 ± 2576.5 ^b^	1266.1 ± 1242.7 ^a^
Pyridoxine (B_6_) ^ii^	92.4 ± 75.8 ^a,b^	115.1 ± 137.3 ^b^	196.7 ± 225.3 ^c^	56.4 ± 60.4 ^a^
Biotin (B_7_)	12.1 ± 9.0 ^a,b^ (95.5%)	14.0 ± 16.5 ^b^ (92.9%)	15.8 ± 20.4 ^b^ (100%)	8.1 ± 6.9 ^a^ (87.8%)
Folic acid (B_9_)	32.3 ± 13.1 ^b^ (53.2%)	58.6 ± 35.5 ^a^ (43.9%)	31.5 ± 1.3 ^a^ (2%)	40.2 ± 19.8 ^b^ (41.1%)
Cyanocobalamin (B_12_)	4.9± 1.1 (13.5%)	5.0± 1.4 (7.1%)	2.7 ± 4 (25.8%)	8.1 ± 4.9 (2.2%)

^i^ Niacin = Nicotinic acid + Nicotinamide. ^ii^ Pyridoxine = Pyridoxal + Pyridoxine. Each value is expressed as mean ± standard deviation. ^a,b^ Means followed by the different letters in a row are significantly different at *p* < 0.05 by Scheffe’s multiple range test. Numbers in parentheses are the percentage of detected samples (concentration > LOQ).

**Table 2:** Average of fat soluble-vitamin concentration in human milk (µg/L).

	CHINA	KOREA	PAKISTAN	VIETNAM
No. Sample	137	254	97	92
Retinol (Vit A)	364.5 ± 232.7 ^a^	356.5 ± 180.1 ^a^	622.1 ± 447.3 ^b^	813.6 ± 609.0 ^c^
Tocopherol (Vit E)	1907.5 ± 1309.1 ^a^	2140.1 ± 896.0 ^a^	3943.0 ± 2874.5 ^b^	4413.4 ± 5274.2 ^b^
Phylloquinone (Vit K)	19.3 ± 16.7 ^a,b^ (68.6%)	18.9 ± 18.5 ^a,b^ (69.7%)	25.7 ± 17.2 ^b^ (62.9%)	12.9 ± 13.9 ^a^ (43.5%)
Lutein	66.1 ± 51.6 ^b^	41.3 ± 27.4 ^a^	47.5 ± 58.9 ^a^	49.7 ± 47.9 ^a,b^

^a,b^ Means followed by the different letters in a row are significantly different at *p* < 0.05 by Scheffe’s multiple range test. Each value is expressed as a mean ± standard deviation. Numbers in parentheses are the percentage of detected sample (concentration > LOQ). Phylloquinone concentration was calculated based on the detected sample only.

**Table 3:** Correlation coefficients between vitamin concentrations in the pooled sample (*n* = 452).

	B_1_	B_2_	B_3_	B_5_	B_6_	B_7_	B_9_	B_12_	Retinol	E	K	Lutein
**B_1_**	1.000											
**B_2_**	0.074	1.000										
**B_3_**	0.318	0.083	1.000									
**B_5_**	0.342	0.181	0.351	1.000								
**B_6_**	0.450	0.362	0.208	0.464	1.000							
**B_7_**	0.137	0.399	0.118	0.228	0.207	1.000						
**B_9_**	0.171	0.115	0.106	0.340	0.165	0.253	1.000					
**B_12_**	0.102	0.098	0.009	0.029	0.007	0.005	−0.005	1.000				
**Retinol**	−0.164	0.012	0.056	−0.068	−0.076	−0.079	−0.091	−0.016	1.000			
**E**	−0.168	0.042	−0.063	−0.109	−0.076	−0.022	−0.089	−0.010	0.497	1.000		
**K**	0.009	0.080	−0.026	−0.034	−0.002	0.067	−0.013	−0.049	−0.065	−0.025	1.000	
**Lutein**	−0.118	−0.039	−0.032	−0.055	−0.084	−0.033	−0.076	−0.095	0.351	0.388	0.081	1.000

**Table 4:** Vitamins and lutein concentration (μg/L) reported in human milk.

Vitamin	Country	Analysis Method	*n*	Median	Mean	SD	Ref.
**Thiamin (Vit. B_1_)**							
	China	UPLC-MS/MS	6419	5.0–40.7			[24]
	Japan	HPLC-FID	691		123	32	[35]
	China	HPLC-MS/MS	443	31.3–62.8			[36]
	Malawian	HPLC-FLD	177	10.5–40.9			[19]
	Bangladesh	HPLC-FLD	18	116			[28]
**Riboflavin (Vit. B_2_)**							
	China	UPLC-MS/MS	6419	29.3–40.6			[24]
	China	HPLC-MS/MS	443	119–208			[36]
	Malawian	ULPC-MS/MS	177	6.3–7.3			[19]
	Bangladesh	HPLC-MS/MS	18	24			[28]
**Niacin (Vit. B_3_)**							
	China	UPLC-MS/MS	6419	470.7–687.0			[24]
	Japan	HPLC-UV	619		329	204	[35]
	China	HPLC-MS/MS	443	1940–3000			[36]
	Bangladesh	HPLC-MS/MS	18	219			[28]
**Pantothenic acid (Vit. B_5_)**							
	China	UPLC-MS/MS	6419	1770.9–2626.8			[24]
	Japan	Microbiological assay	619		2700	900	[35]
	China	HPLC-MS/MS	443	1790–2910			[36]
**Pyridoxine (Vit. B_6_)**							
	China	UPLC-MS/MS	6419	4.6–80.7			[24]
	Japan	Microbiological assay	619		57	25	[35]
	China	HPLC-MS/MS	443	63.4–102.0			[36]
	Bangladesh	HPLC-MS/MS	18	81			[28]
**Biotin (Vit. B_7_)**							
	Japan	Microbiological	619		5.0	2.3	[35]
	China	HPLC-MS/MS	443	4.6–6.1			[36]
**Folic acid (Vit. B_9_)**							
	Japan	HPLC-FID	619		62	29	[35]
	China	HPLC-MS/MS	443	7.3–24.4			[36]
	Canada	HPLC-MS/MS	160		20.7	0.7	[25]
**Cyanocobalamin (Vit. B_12_)**							
	Japan	Microbiological assay	619		0.4	0.2	[35]
	Bangladesh	Quantitative immuno-analyzer	18	0.175			[28]
	India	Competitive protein binding immunoassay	326	0.9–1.8			[37]
**Retinol (Vit. A)**							
	Japan	HPLC-FID	82		455	264	[38]
	Bangladesh	HPLC	18	391			[28]
	Korea	HPLC-UV	334		395.8	196.4	[39]
	Brazil	HPLC-UV	103		624.6	229.2	[40]
	Brazil	HPLC-UV	136		483.3	197.3	[41]
**Tocopherol (Vit. E)**							
	Japan	HPLC-FID	619		3250	1650	[35]
	Japan	HPLC	82		5087	5042	[38]
	Bangladesh	HPLC	18	4400			[28]
	Korea	HPLC-UV	334		230	130	[39]
	Brazil	HPLC-UV	103		11,241.5	5513	[40]
**Cholecalciferol (Vit. D_3_)**							
	Japanese	HPLC-UV	114		0.08		[35]
	Japanese	LC-MS/MS	88		0.088		[38]
	USA	LC-MS/MS	40		0.008–0.04		[42]
	Denmark	LC-MS/MS (LOD: 0.2 nmol)	120		0.11–0.57 (27–46% < LOD)		[43]
**Phylloquinone (Vit. K)**							
	Japan	HPLC-MS/MS	82		3.7	2.2	[38]
	Japan	HPLC-FID			4.3	2.9	[44]
		HPLC-FID		0.9–1.2			[45]
	UK	HPLC-MS/MS	29		2.1–140		[46]
			15		2.87–3.39		[47]
					1.1–130		[48]
**Lutein**							
	Mexico	HPLC-UV	20		27.3	11.8	[49]
	Japan	HPLC-UV	20		29.1	21.3	[49]
	UK	HPLC-UV	20		12.4	7.8	[49]
	China	HPLC-UV	20	93.1			[33]
	USA	HPLC-UV	20	41.7			[33]
	Mexico	HPLC-UV	20	39.2			[33]
	Italy	HPLC-UV	15		62.6	28.4	[34]
	China	HPLC-UV	509	22–58			[50]

The value was converted to the same unit (μg/L).

## Supplementary Materials

The following are available online at https://www.mdpi.com/2072-6643/12/6/1794/s1, Table S1. The quantification channels of the analytes and the parameters used for their quantification. Table S2. The average and median of water soluble-vitamin concentration in human milk (µg/L). Table S3. Correlation coefficients between vitamin concentrations in China sample (*n* = 111). Table S4. Correlation coefficients between vitamin concentrations in Korea sample (*n* = 155). Table S5. Correlation coefficients between vitamin concentrations in Pakistan sample (*n* = 97). Table S6. Correlation coefficients between vitamin concentrations in Vietnam sample (*n* = 90).
