# Supplementary material for: A Comparison of Vitamin and Lutein Concentrations in Breast Milk from Four Asian Countries
Source: Nutrients. 2020 Jun 17;12(6):1794. doi: 10.3390/nu12061794 (PMC7353211; doi:10.3390/nu12061794)
Supplement: Supplementary file 1 [file nutrients-12-01794-s001.pdf]

Supplementary materials

Table S1. The MS/MS parameter and MRM transitions of analytes in positive mode

Analyte	RT (min)	Precursor Ion (m/z)	Product Ion (m/z)	Fragmentor (V)	Collision Energy (V)
Thiamin	8.66	265.1	122.1	97	12
Riboflavin	4.31	377.2	243.1	167	20
Nicotinic acid	3.03	124.1	80.1	107	20
Nicotinamide	1.32	123.1	80.1	112	20
Pantothenic acid	2.28	220.1	90.1	112	20
Pyridoxine	2.00	170.1	152.1	92	8
Pyridoxal	1.65	168.1	150.1	82	8
Biotin	1.37	245.1	227.1	102	12
Folic acid	10.18	442.1	295.2	102	12
Cyanocobalamin	10.59	678.2	147.1	152	40

Table S2. The average and median of water soluble-vitamin concentration in human milk (μg/L)

	CHINA		KOREA		PAKISTAN		VIETNAM	
	Mean±SD	Median	Mean±SD	Median	Mean±SD	Median	Mean±SD	Median
Thiamin (B_1)	67.5±51.4	55.1	89.3±74.3	71.9	66.5±93.7	20.7	56.5±61.5	36.1
Riboflavin (B_2)	20.9±49.6	10.9	30.0±106.4	6.2	58.0±43.3	47.4	15.4±31.7	6.9
Niacin (B_3)[i]	396.7±233.3	329.7	393.7±278.3	366.8	523.9±485.2	371.8	553.8±440.2	406.4
Pantothenic acid (B_5)	1924±2047.4	1364.4	2571.2±2932.2	1387.7	2557.4±2576.5	1372.6	1266.1±1242.7	1041.2
Pyridoxine (B_6)[ii]	92.4±75.8	69.0	115.1±137.3	78.1	196.7±225.3	106.4	56.4±60.4	42.5
Biotin (B_7)	11.6±9.2	9.9	13.0±16.3	8.8	15.6±20.5	8.5	7.1±7.0	6.05
Folic acid (B_9)	17.2±18.8	19.1	25.7±37.4	0.0	0.6±4.5	0.0	16.5±23.5	0.0
Cyanocobalamin (B_{12})	0.7±1.7	0.0	0.4±1.3	0.0	0.7±2.3	0.0	0.2±1.3	0.0

[i]Niacin = Nicotinic acid + Nicotinamide. [ii]Pyridoxine = Pyridoxal + Pyridoxine.

Table S3. Correlation coefficients between vitamin concentrations in China sample (n=111)

	B1	B2	B3	B5	B6	B7	B9	B12	Retinol	E	K	Lutein
B1	1.000											
B2	0.118	1.000										
B3	0.325	0.146	1.000									
B5	0.250	**0.641**	0.309	1.000								
B6	0.330	**0.704**	0.182	**0.618**	1.000							
B7	0.270	0.166	0.178	0.428	0.365	1.000						
B9	0.158	0.023	0.025	0.203	-0.011	0.302	1.000					
B12	0.057	-0.091	0.075	0.021	-0.025	-0.003	0.254	1.000				
Retinol	-0.128	-0.010	0.305	0.045	-0.057	0.066	-0.193	-0.065	1.000			
E	0.010	0.025	0.076	0.023	0.058	0.267	-0.116	-0.103	**0.461**	1.000		
K	-0.017	-0.007	0.043	-0.084	-0.021	-0.099	0.098	-0.010	0.182	0.107	1.000	
Lutein	-0.183	-0.035	0.152	-0.039	-0.061	0.039	-0.337	-0.181	**0.537**	0.443	0.202	1.000

Table S4. Correlation coefficients between vitamin concentrations in Korea sample (n=155)

	B1	B2	B3	B5	B6	B7	B9	B12	Retinol	E	K	Lutein
B1	1.000											
B2	0.099	1.000										
B3	0.292	0.005	1.000									
B5	0.164	0.077	0.540	1.000								
B6	0.327	0.479	0.166	0.285	1.000							
B7	0.260	0.639	0.188	0.351	0.473	1.000						
B9	0.138	0.201	0.223	0.504	0.490	0.403	1.000					
B12	0.221	0.171	0.026	-0.044	0.087	0.089	-0.046	1.000				
Retinol	-0.036	-0.038	0.136	0.155	0.122	0.070	0.209	-0.124	1.000			
E	0.097	0.110	-0.026	0.108	0.103	0.211	0.226	-0.085	0.204	1.000		
K	-0.045	0.063	-0.037	-0.143	-0.040	0.063	-0.069	-0.104	0.059	0.095	1.000	
Lutein	0.073	0.009	-0.046	0.069	0.046	0.083	0.119	-0.096	0.190	0.373	0.150	1.000

Table S5. Correlation coefficients between vitamin concentrations in Pakistan sample (n=97)

	B1	B2	B3	B5	B6	B7	B9	B12	Retinol	E	K	Lutein
B1	1.000											
B2	-0.071	1.000										
B3	0.275	0.204	1.000									
B5	0.549	-0.007	0.291	1.000								
B6	0.621	-0.048	0.229	0.631	1.000							
B7	-0.159	-0.103	0.008	-0.160	-0.159	1.000						
B9	-0.013	0.043	-0.045	-0.112	-0.062	-0.100	1.000					
B12	0.103	0.163	-0.031	0.055	-0.072	-0.065	0.094	1.000				
Retinol	-0.278	0.292	-0.093	-0.182	-0.170	-0.127	0.246	0.086	1.000			
E	-0.288	0.130	-0.219	-0.294	-0.226	0.004	0.168	0.091	0.795	1.000		
K	0.034	0.133	0.106	0.049	-0.100	0.021	-0.090	-0.082	0.026	0.004	1.000	
Lutein	-0.135	-0.132	0.023	-0.065	-0.115	-0.068	-0.090	-0.109	0.660	0.562	-0.058	1.000

Table S6. Correlation coefficients between vitamin concentrations in Vietnam sample (n=90)

	B1	B2	B3	B5	B6	B7	B9	B12	Retinol	E	K	Lutein
B1	1.000											
B2	0.241	1.000										
B3	0.651	0.213	1.000									
B5	0.605	0.357	0.609	1.000								
B6	0.641	0.604	0.488	0.479	1.000							
B7	0.606	0.336	0.450	0.509	0.663	1.000						
B9	0.366	0.331	0.346	0.425	0.395	0.486	1.000					
B12	-0.040	0.054	0.046	0.158	-0.062	-0.135	-0.099	1.000				
Retinol	-0.039	-0.191	-0.121	-0.034	-0.177	-0.110	-0.213	-0.002	1.000			
E	-0.214	-0.081	-0.183	-0.184	-0.258	-0.264	-0.139	-0.030	0.277	1.000		
K	-0.092	0.100	-0.120	-0.088	0.074	0.145	0.051	-0.074	-0.096	0.042	1.000	
Lutein	-0.195	0.042	-0.210	-0.240	-0.194	-0.253	-0.194	-0.036	0.324	0.560	0.106	1.000
